# Blood-Induced Arthropathy: A Major Disabling Complication of Haemophilia

**DOI:** 10.3390/jcm13010225

**Published:** 2023-12-30

**Authors:** Alexandre Leuci, Yesim Dargaud

**Affiliations:** 1 UR4609 Hemostasis & Thrombosis Research Unit, Faculté de Médecine Lyon Est, Université Claude Bernard Lyon 1, 69008 Lyon, France; a.leuci@outlook.fr; 2 Unité d’Hémostase Clinique—Centre de Référence de l’Hémophilie, Hôpital Louis Pradel Hospices Civils de Lyon, 69002 Lyon, France

**Keywords:** haemophilia, arthropathy, joint bleed, ultrasound, MRI, prophylaxis

## Abstract

Haemophilic arthropathy (HA) is one of the most serious complications of haemophilia. It starts with joint bleeding, leading to synovitis which, in turn, can cause damage to the cartilage and subchondral bone, eventually inducing degenerative joint disease. Despite significant improvements in haemophilia treatment over the past two decades and recent guidelines from ISTH and WFH recommending FVIII trough levels of at least 3 IU/dL during prophylaxis, patients with haemophilia still develop joint disease. The pathophysiology of HA is complex, involving both inflammatory and degenerative components. Early diagnosis is key for proper management. Imaging can detect joint subclinical changes and influence prophylaxis. Magnetic resonance imagining (MRI) and ultrasound are the most frequently used methods in comprehensive haemophilia care centres. Biomarkers of joint health have been proposed to determine osteochondral joint deterioration, but none of these biomarkers has been validated or used in clinical practice. Early prophylaxis is key in all severe haemophilia patients to prevent arthropathy. Treatment is essentially based on prophylaxis intensification and chronic joint pain management. However, there remain significant gaps in the knowledge of the mechanisms responsible for HA and prognosis-influencing factors. Better understanding in this area could produce more effective interventions likely to ultimately prevent or attenuate the development of HA.

## 1. Introduction

Haemophilia is an X-linked hereditary disorder characterized by a deficiency in factor VIII (FVIII, haemophilia A) or factor IX (FIX, haemophilia B). Severe haemophilia, defined by factor VIII or IX levels < 1 IU/dL, is associated with the highest risk of spontaneous haemorrhage, in particular joint bleeds, which constitute 65–70% of bleeding events. Several molecular mechanisms make joints the main location of bleeds, such as (i) a very low expression of tissue factor [1]; (ii) high concentrations of tissue factor pathway inhibitor (TFPI) and thrombomodulin, synthesized by chondrocytes and synoviocytes [2,3]; and (iii) increased fibrinolytic activity related to low levels of thrombin-activated fibrinolysis inhibitor (TAFI) induced by an impaired thrombin generation capacity. 

Besides recent significant improvement in haemophilia treatment [4,5], efforts to personalize prophylaxis, using pharmacokinetic tools and adaptation to each patient’s lifestyle and physical activities, have been undertaken [6,7]. These approaches consistently demonstrated the benefits of personalized prophylaxis in reducing joint bleeding. However, despite therapeutic progress enabling more effective prophylaxis for a broader patient population, complications still persist, leading to the morbidity and physical disability caused by haemophilic arthropathy (HA). 

## 2. Pathophysiological Mechanisms of Haemophilic Arthropathy

**Figure 1:**
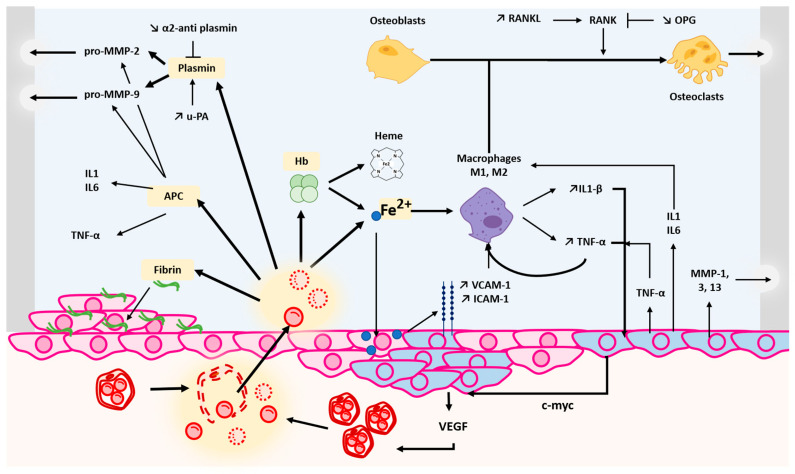
After blood vessel injury in the synovial membrane, blood and blood degradation products such as fibrin, activated protein C, plasmin, haemoglobin and iron are freed inside the articulation capsule. Fibrin deposits on the synovial membrane will be engulfed by proliferating synovial fibroblasts. APC will induce the activation of TNF-α, IL-1 and IL-6 as well as activate pro-MMP-2 and -9. The dysregulation of plasmin activity, downregulation of α2-antiplasmin and upregulation of u-PA will promote plasmin activation toward pro-MMP-2 and -9 activity. Together, pro-MMP-2 and -9 will degrade articular cartilage. On the other hand, haemoglobin will be degraded in haem and iron, increasing iron deposits inside the capsule. Iron accumulation in the synovial membrane will induce an overexpression of adhesion molecules (ICAM, VCAM) that will help recruit macrophages into the synovial fluid. In reaction, macrophages will produce IL1-β and TNF-α. TNF-α itself promotes a macrophage phenotype switch to M1 and M2 in parallel to synovial membrane phenotype switch. Synovial cells will produce TNF-α, IL-1 and IL-6, representing a positive feedback of inflammation inside the articulation. They will also produce MMP1, 3 and 13 leading to cartilage degradation. Inside the capsule, the RANK pathway will also be disturbed with a downregulation of OPG (osteoprotegerin) and upregulation of RANKL (RANK ligand). This will increase osteoclast activity and cartilage degradation. In this context, macrophages represent another pathway to promote osteoclast activity. This inflammation state can ultimately lead to gene dysregulation such as the c-myc gene, promoting hyperplasia and contributing to pannus formation. VEGF is also produced in this context, increasing the risk of blood vessel lesions, thus amplifying and sustaining the whole process towards cartilage degradation and articular destruction. ICAM: InterCellular Adhesion Molecule; VCAM: Vascular Cell Adhesion Protein; pro-MMP2-9: pro-matrix metalloproteinases; IL-1, IL-6: interleukin-1, interleukin-6; RANK: Receptor Activator of Nuclear Factor κ B; APC: Activated Protein C; Hb: haemoglobin; Fe^2+^: iron (II); u-PA: urokinase-type plasminogen activator; OPG: osteoprotegerin.

The pathogenesis of HA is complex and involves several critical steps (Figure 1) [8,9].

### 2.1. Joint Bleeding

Joint bleeding occurs mostly in weight-bearing joints such as the knees, ankles and elbows [6,10,11]. Besides blood vessel injuries in the synovium, responsible for joint bleeding [8], such breakdown products as iron, haemoglobin, fibrin, plasmin and activated C protein (ACP) favour inflammation and ultimately synovitis. Consequently, the synovium becomes thicker and produces an excess of synovial fluid, thus contributing to joint swelling and pain [9].

### 2.2. Effects of Blood Breakdown Products on Joint Health

#### 2.2.1. Iron Deposition

Following joint bleeds, iron from haemoglobin will accumulate in the synovium [12,13,14], together with haemosiderin, an iron-storage protein. Haemoglobin is then degraded by CD163 into globin and haem (which contains iron), or taken-up by the haem carrier protein HCP1. Iron liberated from the haem can by phagocytosed by macrophages or bound by ferroportin. Saturation of the latter leads to an excess of iron deposition that can amplify iron toxicity through the production of free radicals [15,16,17]. As shown in hereditary haemochromatosis arthropathy, the overexpression of VCAM-1 could participate in iron deposition in HA by contributing to the recruitment of immune cells [18]. In haemophilic mice presenting blood-induced joint damage, the NFk-B pathway has also been shown to be upregulated, leading to hypoxia-induced factor (HIF)-2α overexpression [19]. HIF-2α upregulates nitric oxide synthase 2, which results in an accumulation of nitric oxide (NO) and induces a downregulation of the production of reactive oxygen species (ROS) [20,21]. HIF-2α can also upregulate catabolic proteins such as matrix metalloproteinases (MMPs) [8,10,15,18,19,22].

#### 2.2.2. Fibrinolysis Proteins

Urokinase plasminogen activator (u-PA) and plasmin are upregulated in the synovial tissue in arthropathic haemophilia mice, compared to haemophilia mice [23]. Plasmin is a serine protease able to activate pro-MMP-2 and -9, leading to plasmin-induced cartilage degradation. In addition, the upregulation of plasmin formation by u-PA and the defective downregulation of fibrinolysis, shown by an absence of upregulation of α-2-antiplasmin, could amplify plasmin lysis activity [24]. According to Nieuwenhuizen et al. [25], targeting u-PA effectively leads to a decrease in synovial u-PA but is still less effective for reversing HA than targeting plasmin. This could mean that plasmin is the major component of the fibrinolytic pathway inducing HA, and not its canonical regulator (i.e., u-PA). Fibrin deposition has also been reported to induce synovitis, as shown by Sanchez-Pernaute et al. [26].

#### 2.2.3. Activated Protein C

APC, an important natural anti-coagulant protein, prevents excessive blood clot formation by inactivating factors V and VIII [27]. APC is also associated with an increase in MMP-2 and MMP-9 activity in the joints [28]. In haemophilic mice, an inhibition of APC induces IL-1β and IL-6 downregulation, a decrease of TNF-α and vascular leakage [29,30]. Therefore, inhibiting APC could be a therapeutic method to decrease inflammation in the onset on HA. 

### 2.3. Synovial Inflammation

As part of the inflammatory response, blood vessels in the synovium dilate, leading to an increase in blood flow in the joint. This influx of blood and inflammatory cells exacerbates the inflammatory process. The release of pro-inflammatory molecules from macrophages, such as cytokines (e.g., interleukin-1β [IL-1β] and tumour necrosis factor-alpha [TNF-α]) [12,19], maintains inflammation and helps recruit immune cells into the joints. The synovial membrane responds to this inflammation by producing an excess of synovial fluid, which accumulates in the joint. This fluid contributes to joint swelling, distension and pain.

### 2.4. Consequences of Recurrent Bleeding Episodes

In individuals with haemophilia, the joint’s limited capacity to self-repair is further hampered by recurrent bleeding episodes. These bleeds can occur even with minimal trauma or spontaneously, leading to progressive joint damage. In cases of recurrent joint bleeding, synovial inflammation becomes persistent and chronic. In this inflammatory environment, synoviocytes increase their production of pro-inflammatory cytokines such as IL-1, IL-6 and TNF-α. It has been shown that injection of IL-4 and IL-10 [31] can prevent blood-induced cartilage degeneration by suppressing cytokine release from macrophages. This confirms the pivotal role of cytokines in perpetuating the inflammatory response within joints. The inflammatory state is also responsible for the overexpression of adhesion molecules such as VCAM-1 and ICAM-1 on altered synoviocytes [18]. These molecules mediate the attachment and infiltration of inflammatory cells into the synovium as shown by the increase in M1 and M2 macrophages in the joint [32]. 

#### 2.4.1. Pannus Formation

In severe cases, synoviocytes can undergo a transformation into a fibroblast-like phenotype, associated with an increased production of extracellular matrix components and fibrotic changes within the joint, known as pannus formation. Pannus is an abnormal layer of granulation tissue that can invade and damage joint structures, including cartilage and bone [33]. It contributes to the progression of joint degeneration. Chronic inflammation and bleeding lead to an increased number and hypertrophy of synoviocytes [12,34]. This is often associated with synovial thickening and is a hallmark of synovitis in HA. 

#### 2.4.2. Angiogenesis Induction 

In response to chronic inflammation, synoviocytes can promote angiogenesis mediated by Vascular Endothelial Growth Factor (VEGF). A 10-fold increase in VEGF is observed within the synovial membrane [35,36,37] in HA joints, while no VEGF is found in the plasma of patients. The newly created blood vessels maintain the continuous blood leakage within affected joints [37].

#### 2.4.3. Cartilage and Bone Degradation

Prolonged exposure to blood within the joint space can lead to cartilage and bone damage. The iron and haemosiderin released by damaged red blood cells can lead to a progressive loss of cartilage, cartilage erosion and bone destruction within the affected joint. Synoviocytes can upregulate the expression of MMP-1, MMP-3 and MMP-13 [8,32]. These enzymes are responsible for degrading extracellular matrix components, including collagen and proteoglycans, thus contributing to cartilage degradation. In the context of HA, increased levels of RANK and RANK ligand (RANK-L) in the synovium are associated with a decreased level of osteoprotegerin (OPG). RANK-L binds to its receptor, RANK, present on the surface of osteoclasts. OPG can also bind to RANK-L, preventing its interaction with RANK. An unbalanced RANK-RANKL-OPG axis promotes the activation of osteoclasts [38,39]. The ongoing cycle of bleeding, inflammation, cartilage degradation and bone resorption perpetuates the joint damage seen in HA. Over time, this can result in chronic joint pain, swelling and reduced joint mobility.

## 3. Risk Factors for Haemophilic Arthropathy

Arthropathy is more common in patients with severe haemophilia [40], but is also observed in patients with moderate or mild haemophilia [41]. Prophylaxis can, however, significantly reduce the risk of HA. Two prospective, randomized, controlled clinical studies [42,43] have demonstrated an 80–85% reduction in joint bleeds among children receiving primary prophylaxis.

Another prospective study [44], involving haemophilia A patients with arthropathy on secondary prophylaxis for 3 years, showed that secondary prophylaxis stabilizes joint damage but may not allow its regression. This finding demonstrates the irreversible nature of established joint damage and emphasizes the crucial role of primary prophylaxis for joint protection. Previous studies have indicated that personalized prophylaxis using pharmacokinetics (PK) tools can enhance the effectiveness of prophylaxis by tailoring it to individual patient characteristics, achieving equivalent factor concentrate consumption and cost-effectiveness [45]. However, the minimum residual FVIII/IX level required for complete protection against arthropathy remains uncertain. The introduction of extended half-life molecules and non-factor therapies made it easier to achieve higher trough FVIII/IX levels. This significant advancement in therapies has led to new recommendations from the World Federation of Haemophilia (WFH), which now recommends residual levels of 3 to 5 IU/dL (as opposed to the historical > 1 IU/dL) during prophylaxis to reduce more effectively the risk of joint bleeds and HA [46]. However, plasma FVIII/IX levels are not the sole determinant of a clinical bleeding phenotype. Several studies have reported that the overall coagulation effectiveness, as assessed through thrombin generation capacity, can better reflect the individual bleeding risk compared to the assessment of FVIII or FIX activity [47]. An international multicentre prospective clinical study confirmed that the ability to generate thrombin correlates better with the risk of bleeding than FVIII levels in haemophilia A [48]. Recent research has facilitated the integration of pharmacokinetics and pharmacodynamics to optimize the personalization of prophylactic regimens using FVIII concentrate [49]. 

The development of neutralizing anti-haemophilic alloantibodies, or inhibitors, is a primary complication in haemophilia treatment. This occurrence increases the risk of bleeding and accelerates joint damage [50]. Additionally, the immune response against FVIII can exacerbate the inflammatory reaction in joints [51]. Consequently, haemophilia patients with inhibitors face a higher risk of HA compared to those without inhibitors.

Genetics also plays a significant role in the risk of HA, primarily through mutations affecting FVIII/IX levels and increasing the risk of inhibitors [51]. Furthermore, mutations in pro-inflammatory proteins can influence individual susceptibility to HA. The TNFα-308 G > A mutation is thus frequently associated with the innate immune response and the development of subchondral cysts in haemophiliacs [52]. This susceptibility can be explained by the activation of innate immunity through Toll-like receptor 10 (TLR10) [52]. A multicentre study involving children and adolescents with haemophilia identified 613 single-nucleotide polymorphisms (SNPs) correlated with joint mobility [53]. Several mutations linked to the development of HA have been observed in haemophiliacs, highlighting the role of genetics in joint prognosis.

Local risk factors at the joint level can also contribute to the development of HA. Mechanical stress, particularly at the ankles and knees, due to body weight bearing, increases the risk of arthropathy [54]. In addition, chronic synovitis induces an increase in the production of pro-fibrinolytic molecules by synoviocytes. The local rise in plasmin levels exacerbates joint susceptibility to haemorrhage [23].

It has been suggested that uPA could directly contribute to cartilage and bone degradation, independently of its fibrinolytic action [23]. In line with these data, injecting a plasmin-inhibiting substance in the joints reduces cartilage degradation in a mouse model of joint bleeding [55]. Another study demonstrated the beneficial effect of tranexamic acid on joint bleeding risk in a haemophilia A mouse model receiving prophylaxis with FVIII concentrate or emicizumab [56]. Mice receiving combined prophylaxis with FVIII + tranexamic acid or emicizumab + tranexamic acid developed fewer joint bleeds than those receiving traditional prophylaxis with FVIII or emicizumab alone. 

Several environmental factors increase the risk of HA, including overweight and obesity, which are associated with more frequent joint bleeds. A study involving both adult and paediatric haemophiliacs found a 10% higher incidence of haemarthroses in overweight or obese patients (34.4% vs. 24.6% in children and 46.3% vs. 38.6% in adults) [57]. The same study also noted reduced ankle and knee joint mobility in obese haemophiliacs compared to those with a normal weight. 

A healthy lifestyle, including regular physical activity, plays a crucial role in protecting joints. Many individuals with severe haemophilia tend to be less physically active due to mobility challenges, pain and fear of bleeding incidents. Several studies have reported that physical activity not only strengthens muscles, providing better joint stability, but also enhances bone mineralization and bone mineral density [58,59]. In cases where prophylaxis is not used, severe and moderate haemophiliacs face a higher risk of bleeding incidents during physical activities compared to those with mild haemophilia. It has been shown that a 1 IU/dL increase in FVIII/IX levels reduces the risk of bleeding events by 2% [60]. Additionally, physical activity helps maintain the muscle-to-fat ratio and combats obesity, an independent risk factor for HA, especially in joints subjected to mechanical stress such as ankles [61]. It has been suggested that the buildup of lactic acid during anaerobic exercise might affect FVIII clearance and lead to increased circulating FVIII levels [62]. Beyond the physical benefits for muscles, bones and joints, sports offer significant psychosocial advantages to individuals with haemophilia [63]. With current therapeutic advancements achieving low annual bleeding rates, patients with haemophilia have an excellent opportunity to engage in regular and adapted physical activities.

Osteopenia and osteoporosis are additional factors that worsen the progression of HA. A study conducted with adult haemophiliacs, averaging 45.6 years in age and primarily treated on-demand, found osteopenia rates ranging from 28% to 69.5% [64].

It is worth noting that both FVIII and thrombin play roles in bone metabolism [65]. As mentioned above, the activation of osteoclasts, responsible for bone resorption, involves a signalling pathway mediated by RANK, RANK-L and OPG. FVIII, along with its chaperone molecule, von Willebrand factor, can bind to RANK, inhibiting this signalling pathway and, in turn, the maturation and activation of osteoclasts. FVIII thus plays a protective role against bone resorption. Additionally, thrombin interacts with osteoblasts, responsible for bone synthesis, through their PAR-1 receptors. Reduced thrombin generation in patients with haemophilia A or B can negatively impact bone formation. While it is not clear whether the effect of FVIII or thrombin is predominant in regulating bone metabolism, it is obvious that the combined impact of these two physiological regulation pathways in individuals with severe haemophilia can lead to osteopenia.

This predisposition to osteoporosis is further exacerbated in certain patients who are exposed, either frequently or for extended periods, to corticosteroid treatments that weaken their bones. Another factor contributing to osteopenia and osteoporosis is the presence of such comorbidities as HIV or hepatitis C infections and their associated treatments [66].

## 4. Diagnosis of Haemophilic Arthropathy

**Figure 2:**
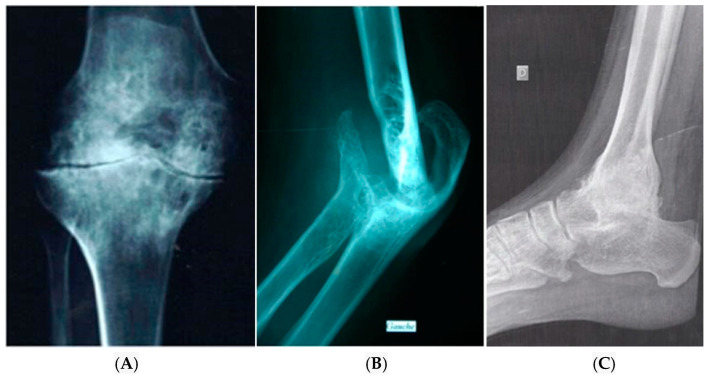
Severe arthropathy of the knee (**A**), elbow (**B**) and ankle (**C**): (**A**) shows subchondral cysts of various size, joint space compression, cartilage destruction, articular surface irregularities and changes in the joint axis. (**B**) shows a destroyed elbow joint and (**C**) shows spontaneous ankylosis of the ankle. Gauche: left, D: right.

The diagnosis of HA relies on radiological assessments. Historically, bone radiography was the primary diagnostic tool, enabling the identification of arthropathy in advanced cases. The radiological Petterson score was used to grade the severity of joint damage [67]. Key radiological signs include oedema of peri-articular soft tissues, epiphyseal enlargement, loss of articular cartilage, joint space narrowing, bone erosion, the formation of subchondral cysts and geodes, as well as the deformation of bone articular surfaces with irregularities that can progress to depression of the articular surfaces and changes in the articular axis (Figure 2). 

This imaging technique is not ideal for an early detection of HA but serves well for monitoring known arthropathies and is a part of orthopaedic assessments, aiding in the discussion of surgical interventions.

Computed tomography (CT) is a powerful tool for the detection of bone lesions, but it is not suitable for screening synovial abnormalities. Moreover, it is associated with high levels of radiation, which limits its repeated use for monitoring bone lesions. Magnetic resonance imaging (MRI) is thus considered the gold standard for the early diagnosis of HA. It enables the visualization of early synovial damage, including haemosiderin deposition [68]. MRI also allows for a precise analysis of cartilage and subchondral bone changes. Two standardized MRI scoring systems have been developed to assess the severity of joint damage, the Denver MRI score [69] and the score developed by the International Working Group on Prophylaxis [70].

**Figure 3:**
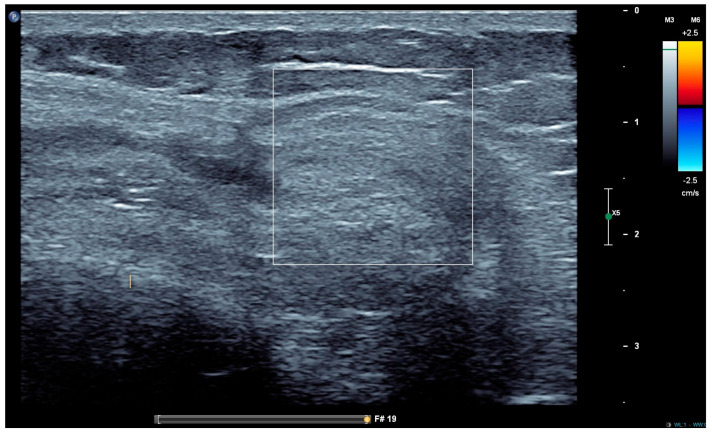
Chronic synovitis with major thickening of the synovium.

Despite the excellent performance of MRI, it is not routinely used due to several barriers. These include limited the availability, high costs and the challenges of implementing it in children, particularly the need for sedation or even anaesthesia in the youngest patients. While MRI is the best method for early joint damage detection in haemophiliacs, it is interesting to note that no correlation has been observed between joint damage shown by MRI and the number of annual joint bleeds [44]. This observation suggests that “asymptomatic microbleeds” may be responsible for joint damage. Recently, joint ultrasound was included in the diagnostic strategy of HA, using standardized procedures. Thanks to these protocols, joint ultrasound has become a routine examination used in haemophilia centres as part of the usual monitoring of patients. The main advantages of ultrasound are its non-ionizing nature, ease to perform, low cost, lack of induced pain and good performance for the early detection of joint damage. Indeed, ultrasound has several potential applications. It can be used for the diagnosis and monitoring of haemarthroses, as it is able to detect the presence of very low amounts of blood (5%) within the synovial fluid [71]. Ultrasound also has a good sensitivity for the detection of synovial hypertrophy, osteochondral changes and superficial bone lesions (Figure 3).

Several standardized protocols have been developed for the assessment of joint status in haemophiliacs. The HEAD-US method is strongly correlated to MRI and is the most widely used method in haemophilia centres [72,73]. One of the primary limitations of ultrasound is the challenge of performing and interpreting it in children, who have thick and immature cartilage, ongoing growth and age-dependent ossification centres. Moreover, ultrasound results are highly operator-dependent.

Clinical monitoring of the joints has been standardized using the validated clinical score from the International Society of Thrombosis and Hemostasis (ISTH). Known as the HJHS (Hemophilia Joint Health Score), it provides a global functional assessment of the joints [74].

Laboratory assays are not yet integrated into the diagnostic toolkit for HA. There are studies on serum or urine biomarkers of joint damage in haemophiliacs [75], but the correlation between these biomarkers and joint prognosis is minimal, likely due to the diversity of the populations studied and study designs. Further prospective clinical studies are needed to elucidate the potential role of joint biomarkers in the early-stage diagnosis of HA complications.

## 5. Treatment of Haemophilic Arthropathy

The prevention of HA is primarily based on the substitution of the deficient factor to avoid joint bleedings. However, treatment methods remain extremely limited. Notably, none of the currently available treatments specifically targets synovitis, which represents the early and reversible stage of joint damage. Various therapeutic approaches, complementary to anti-haemophilic treatment, exist, and some are currently under evaluation. These approaches include early aspiration of intra-articular blood following haemarthrosis, intra-articular corticosteroid infiltration, embolization, chemical synoviorthesis (using rifampin or tetracycline), radioisotope treatment, surgical synovectomy, intra-articular injection of platelet-rich plasma (PRP) and intra-articular injection of hyaluronic acid [76]. 

After the onset of synovitis, treatment with FVIII/IX concentrates or non-substitute therapies alone is insufficient to avoid joint damage. Currently, no anti-inflammatory treatment, such as anti-TNFα, methotrexate, hydroxychloroquine or other Disease-Modifying Anti-Rheumatic Drugs (DMARDs), have been evaluated for their effectiveness in managing HA [77]. In some cases, intra-articular corticosteroid infiltration may provide temporary relief for selected patients, but the systematic benefit of corticosteroid infiltration has not been conclusively demonstrated by clinical studies with appropriate methodologies [78]. 

Recently, the effectiveness of intra-articular injections of PRP or hyaluronic acid, both of which are already used in other rheumatological conditions, has been investigated [79]. This showed that, in the presence of growth factors from the PRP administered to an inflamed joint, synovial fibroblasts increase their production and secretion of hyaluronic acid and VEGF, which could enhance hyaluronic acid levels and better regulate angiogenesis in joints affected by HA [80].

Chemical synoviorthesis using rifampicin or tetracycline as well as radioisotope synovectomy or embolization are methods that can help limit the progression of chronic synovitis and alleviate the pain and reduced joint mobility associated with this condition. This treatment is primarily targeted at patients with synovial hypertrophy, with reported success rates of up to 80% improvement in clinical symptoms. Surgical synovectomy shares similar indications to chemical synoviorthesis. 

The only treatment that has demonstrated Its effectiveness is prosthetic orthopaedic surgery, which is typically reserved for advanced stages of HA, a condition responsible for physical and social disability. The results of joint prostheses can be improved through post-operative rehabilitation and pre- and post-operative muscle strengthening. A recent meta-analysis of 28 surgical studies, encompassing 1210 total knee prostheses in 917 adult patients with haemophilia of a mean age of 38.5 ± 5.1 years, and a mean follow-up of 7.1 ± 2.9 years, reported a significant improvement in joint mobility by 22.3 degrees. Complications were relatively infrequent, with 19.3% cases requiring surgical revision, 6.2% infections and 7.2% haemarthroses [81]. In certain cases, surgical arthrodesis can help patients regain the use of joints adjacent to the operated joint while reducing pain. However, it is important to note that this type of surgery is not intended to improve the mobility of the affected joint.

Several promising therapeutic approaches are being explored to repair damaged articular cartilage and are currently in pre-clinical development using animal models. These approaches include the administration of messenger RNA of cartilage anabolism transcription factors [82] and cartilage engineering based on alginate hydrogel or mesenchymal stem cells [83]. Additionally, a pilot clinical study is underway to assess the effectiveness and safety of an anti-angiogenic (anti-VEGF) medication, bevacizumab (Avastin^®^, Roche, Basel, Switzerland), in preventing HA (NCT 02060305).

## 6. Prevention of Haemophilic Arthropathy: A Challenge for Today and Tomorrow

The primary goal of anti-haemophilic prophylaxis is to reduce and prevent joint bleeds and preserve joint health [84]. A study using data from the British Haemtrack registry, which involved 273 patients with haemophilia A or B, both adults and children, examined the occurrence of haemarthrosis during prophylaxis over one year [85]. Among haemophilia B patients, 82% of children and 68% of adults received prophylaxis with an extended half-life FIX product, compared to 33% of children and 23% of adults with haemophilia A. The study documented 2238 haemarthroses, with two-thirds of children and one-third of adults on prophylaxis experiencing no joint bleeding. In the paediatric population, the median HJHS score was zero, while in adults, ankles were the most affected joints (median HJHS 4 [0–8]). These findings align with the higher prevalence of arthropathy observed in ankles compared to other joints [86]. 

## 7. Management of Haemophilic Arthropathy with a Personalized Medicine Approach

In 2023, at comprehensive haemophilia care centres with multidisciplinary teams, the approach to fight arthropathy is based on (i) early prophylaxis, sometimes personalized, using pharmacokinetic tools; (ii) therapeutic education with the goals of enhancing compliance with prophylactic treatment, enabling patients to self-treat during joint bleeding and preventing specific bleeding incidents through appropriate behaviour; (iii) tailored physical activity; (iv) screening for and management of osteopenia; and (v) careful follow-up of joints with ultrasound and/or MRI.

The combination of these various tools makes it possible to significantly reduce the risk of debilitating HA, although it may not completely eliminate the risk of joint damage. After achieving the “zero spontaneous bleeding” objective, thanks to the development of new anti-haemophilic medications, progressing toward the “zero arthropathy” goal will require a shift from a “one-size-fits-all” approach towards personally tailored prophylaxis in the era of artificial intelligence.

## 8. Conclusions

HA continues to be a challenging complication of haemophilia, even with well-administered prophylaxis using increasingly effective anti-haemophilic medications. A more comprehensive grasp of the underlying mechanisms, alongside improved imaging and biological techniques, combined with personalized care tailored to each patient’s specific needs, may provide a more effective means to address this significant complication responsible for physical disability in individuals with haemophilia.

## Data Availability

Not applicable.
